# Travelling Wave Pulse Coupled Oscillator (TWPCO) Using a Self-Organizing Scheme for Energy-Efficient Wireless Sensor Networks

**DOI:** 10.1371/journal.pone.0167423

**Published:** 2017-01-05

**Authors:** Zeyad Ghaleb Al-Mekhlafi, Zurina Mohd Hanapi, Mohamed Othman, Zuriati Ahmad Zukarnain

**Affiliations:** Department of Communication Technology and Network, Faculty of Computer Science and Information Technology, Universiti Ptura Malaysia, UPM 43300, Selangor, Malaysia; West Virginia University, UNITED STATES

## Abstract

Recently, Pulse Coupled Oscillator (PCO)-based travelling waves have attracted substantial attention by researchers in wireless sensor network (WSN) synchronization. Because WSNs are generally artificial occurrences that mimic natural phenomena, the PCO utilizes firefly synchronization of attracting mating partners for modelling the WSN. However, given that sensor nodes are unable to receive messages while transmitting data packets (due to deafness), the PCO model may not be efficient for sensor network modelling. To overcome this limitation, this paper proposed a new scheme called the Travelling Wave Pulse Coupled Oscillator (TWPCO). For this, the study used a self-organizing scheme for energy-efficient WSNs that adopted travelling wave biologically inspired network systems based on phase locking of the PCO model to counteract deafness. From the simulation, it was found that the proposed TWPCO scheme attained a steady state after a number of cycles. It also showed superior performance compared to other mechanisms, with a reduction in the total energy consumption of 25%. The results showed that the performance improved by 13% in terms of data gathering. Based on the results, the proposed scheme avoids the deafness that occurs in the transmit state in WSNs and increases the data collection throughout the transmission states in WSNs.

## Introduction

The rising research trend in the area of micro-electrical mechanical systems (MEMS) over the years has provoked scholarly attention focused on WSNs. WSNs can be described as small and inexpensive devices with sensing, processing and transmitting capabilities concerning environmental phenomena of interest. They have various application prospects, including use in military, industrial and agricultural monitoring systems [1]. In a network of sensor nodes, the wireless sensors collaboratively sense the environment, detect the phenomenon of interest, and eventually forward the data to a dedicated base station in synchronization. Among the models that have been extensively investigated in recent years for such collaborative synchronization in WSNs is the firefly-inspired scheme for travelling waves [2]. This synchronization between the nodes is required for coordinating power cycles, energy efficiency and stable functioning of sensors in real-time monitoring scenarios [3].

One of the techniques used for modelling the WSN behavior is the PCO algorithm. This algorithm has been previously utilized to model the flashing light emission attracting mating partners [3–5]. Nevertheless, WSNs are unable to afford the simultaneous transmission and reception of data. Moreover, for most scenarios, battery replacement is impossible upon the exhaustion of a node’s energy. Thus, energy-efficient protocols represent vital design requirements for the PCO in WSNs [6]. Another important requirement of WSNs is the self-organizing capability. This feature enables the sensor nodes to re-discover their new neighbours (due to battery exhaustion or the abrupt malfunction of some nodes in the network) when subject to dynamic network topology changes. Energy efficiency is the main concern in designing sensor nodes because of the limited and non-rechargeable power resource [3]. Therefore, the objective of the proposed scheme is to utilize phase locking of the PCO model to mimic the flashing synchronization behaviour of fireflies to solve the deafness problem among sensor nodes. It is also intended to improve the capability of data gathering of the WSN and minimize the energy consumption of the network. The remainder of this paper is organized as follows: Section 2 reviews previous related work. Section 3 provides an in-depth illustration of the proposed scheme in this study, and Section 4 underscores the performance evaluation of the proposed method. A summary of the work reported in this paper is provided in Section 5.

## 1 Related Work

This section reviews the background of travelling waves based on the phase locking of the PCO models for WSNs. PCO is used to explain the synchronous behaviours of biologically inspired network systems that are divided into three-part pacemaker cells, as observed in the flashing synchronization behaviours of fireflies and neurons [7, 8]. However, the review of this paper concentrates on the PCO model, which is based on the synchronous flashing of the synchronization behaviors of fireflies. This model utilizes the firefly synchronization behaviour used to attract mating partners [5, 9]. However, the PCO model is not suitable for sensor networks given that the WSN nodes are usually unable to receive data packets while transmitting them due to deafness [4, 5]. This problem is generally addressed by adopting the PCO model based on travelling waves, as observed in flashing fireflies as firing.

Another important research consideration in WSNs is energy efficient. In WSNs, energy efficiency is ensured by explicitly embedding energy minimization protocols into the underlying sensing model of the sensors (such as reducing the per packet energy consumption) or avoiding the high energy usage of any single node within the network [6, 10–13]. Therefore, the energy-efficient transmission scheduling for time synchronization on WSNs is classified into a sender and a receiver [11]. The transmission scheduling problems at the sender, which is the focus of the current study, are collision, deafness, and hidden terminals. The problems related to the transmission scheduling at the receiver are idle listening and overhearing.

Mimicking the behaviour of fireflies can facilitate the development of WSNs. Fireflies resemble sensor nodes in terms of their decentralized behaviours, which are characterized by restricted individual processing capabilities [3]. In addition, communication by fireflies is described as largely local. Firefly models that are biologically inspired are classified into mathematical models and biological models [14, 15]. As reported in previous research, the applications of these models have been documented in a variety of WSN works for inter-node communication. In [12], the researchers proposed a multichannel protocol for high-bandwidth WSNs by combining TDMA and frequency-division multiple access. This protocol was intended to overcome the problems of deafness and packet collision scheduling of transmissions. In this regard, the same researchers used reinforced learning for collaborative scheduling and routing in each node in conjunction with service quality (i.e., packet delivery ratio, end-to-end latency, and energy waste factor). This study could address the anticipated conditions easily and frequently with minimal latency and packet loss. As reported by those researchers, the developed protocol was capable of achieving improved operations related to end-to-end delivery rate and end-to-end latency and enhancing the energy efficiency compared to other protocols.

Robert and Wilfried [5] suggested a self-organizing principle based on biological fireflies and used the PCO model for the synchronous emission of light flashes with the objective of attracting mating partners as well as distributing the timing of light flashes in a particular time window. This can avoid deafness without affecting the quality of synchronization. Their study did not require a dedicated synchronization node, and therefore, no single point of failure occurred. In addition, it was found that the additional rate calibration scheme permits a longer interval of resynchronization and the use of inexpensive oscillators with high drift rates as features for low-cost sensor nodes. Furthermore, a synchronization precision of lower than 1 ms is possible to achieve in this scheme. In [4], the researchers first developed a self-organizing mechanism that relied on phase-locking in PCO, aimed at propagating sensor node data from the rim of the network to the sink at a certain hop count for the purpose of avoiding deafness. A simple random-based scheme and a desynchronization-based scheme was proposed for the center of the anti-phase in the PCO model. The scheme is used to overcome the problem of collisions among sensors while maintaining the same hop count that is obtained under the same energy efficiency ratio and data-gathering ratio. The desynchronization-based mechanism developed by those researchers suits the applications of WSNs that require a high level of data collection as well as an efficient and clear scheme instead of simply a high data-gathering ratio. The authors in [7] developed a self-organizing communication scheme and a fully distributed for WSNs that focused on investigating initial conditions that result from creating a desirable shape of the travelling wave regardless of the initial phase of the oscillators in a PCO scheme. Their suggested scheme was found to be capable of collecting information according to the requirements of applications in a dynamic WSN and of delivering sensor node information from/to appointed sensor nodes in an energy-efficient manner compared to [16], even though it takes time to generate a travelling wave. The researchers in [17] proposed a network synchronization method that was developed based on the PCO model. This proposed method indicates that the PCO can be a reasonable option for classic MAC policies, and the method plays a role in motivating additional research into investigating cooperative communication.

In [18], the authors presented a stepwise synchronization-based inter-networking method based on the PCO model to achieve stepwise synchronization separately and obtain smooth and moderate inter-networking between WSNs with diverse operational frequencies. Their studies did not evaluate such scenarios in which there are more than three networks to cooperate and in which the degree of and the number of border nodes overlapping change dynamically. In [19], the authors presented an evolutionary game theory-based channel access mechanism, on this basis, a channel access mechanism based on evolutionary game is proposed to solve maximization problem of system throughput for multiple bounded rationality users sharing multiple channels in WMSN. Their studies did not design new reward function and evaluate network performance from another perspective. The researchers in [20] reviewed efficient routing algorithms for preserving k-coverage in a sensor network and then proposed an effective technique for preserving k-coverage and the reliability of data with logical fault tolerance. Their studies showed that the proposed method provides greater efficiency energy consumption. In [21], the authors proposed a new technique to organize the advanced nodes and to select the CHs in WSNs. Their studies did not focus on transfer protocol that comprises the energy and performance saving, and implement high-power sensors as a gateway between the CH and FN of Fog architecture. The researchers in [22] addressed energy efficiency issues by proposing a novel deployment scheme. This scheme, introduces a hierarchical network design; a model for the energy efficient IoT; and a minimum energy consumption transmission algorithm to implement the optimal model. Their studies did not exploit the benefits of heterogeneity and also propose improvements of end to end delay, data compression techniques, packet delivery ratios and throughput parameters, to achieve a more efficient green IoT.

The above investigations of previous research have enriched our understanding of distinguishing among communication phases, acquisition and network synchronization, where the data are transmitted and received. However, the above proposed schemes in previous research are not suited for applications of WSNs because WSNs are subject to changes in topology.

## 2 Proposed Algorithm

As mentioned previously in Section 1 and Section 2, WSNs are subject to strict energy requirements. Therefore, the proposed technique must cater to these dynamics to achieve a successful implementation, which will subsequently reduce the energy consumption rate and increase the data-gathering rate to avoid the deafness problem. This requires extending the lifespan of the sensor nodes while satisfying the power consumption and energy-efficiency requirements simultaneously. The proposed TWPCO uses the flashing synchronization behaviour of fireflies, where the emission of radio signals represents firing. This process will be triggered in the case where the sensor has a data packet that needs to be transmitted to the base station during the transmission scheduler at the sender state. TWPCO architecture firefly models, which are biologically inspired, are generally grouped into mathematical models [23–25] and biological models. Such models have been utilized in a variety of WSN applications, particularly for inter-node communication [15, 26].

### 2.1 TWPCO Mathematical Model

The PCO technique [23, 24, 27] involves the synchronization behaviour of a number of oscillators, in which an oscillator only fires when its timer reaches 1. The traditional PCO technique describes three patterns of synchronous firefly behaviour: in-phase, anti-phase, and phase-locking, according to the PCO synchronization. The in-phase behaviour based on oscillators performs complete synchronization. The anti-phase behaviour based on oscillators performs synchronization with an equal interval. The phase-locking behaviour based on PCO synchronization involves the synchronization of the oscillator with a constant offset.

Given a set of *N* oscillators *ϕ*_*i*_, where 1 ≤ *i* ≤ *N*, each oscillator *ϕ*_*i*_ is associated with a phase *ϕ*_*i*_ (such that *ϕ*_*i*_ *ϵ*[0, *T*]). Over time, *ϕ*_*i*_ is bound to shift towards *T* (which is the maximum). At *T*, the oscillator *ϕ*_*i*_ fires before *ϕ*_*i*_ returns to zero. Similarly, the oscillator *ϕ*_*j*_ that is paired with the firing oscillator *ϕ*_*i*_ is stimulated. This moves the corresponding phase *ϕ*_*j*_ by an infinitesimal amount Δ(*ϕ*_*j*_), where *ϕ*_*j*_ = Δ(*ϕ*_*j*_)+*ϕ*_*j*_.

The total Δ(*ϕ*_*j*_) is given by

$$\begin{array}{c} \phi_{j}=\Delta \left(\phi_{j}\right)+\phi_{j} \end{array}$$ (1)

where Δ(*ϕ*_*j*_) denotes the PRC, which is widely used by experimentalists to quantify the behaviour of a system without knowing the underlying mechanisms responsible for the behaviour [24, 28–31]. The PCO firing is presented by adopting the travelling wave equation based on phase-locking of the PCO model.

**Fig 1:**
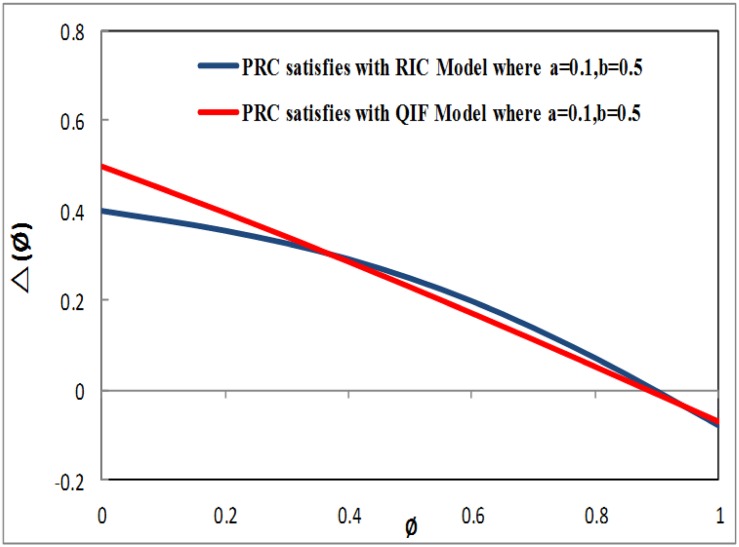
Evaluation of PRC (Δ_*s*_(*ϕ*)) with RIC model and QIF model.

**Fig 2:**
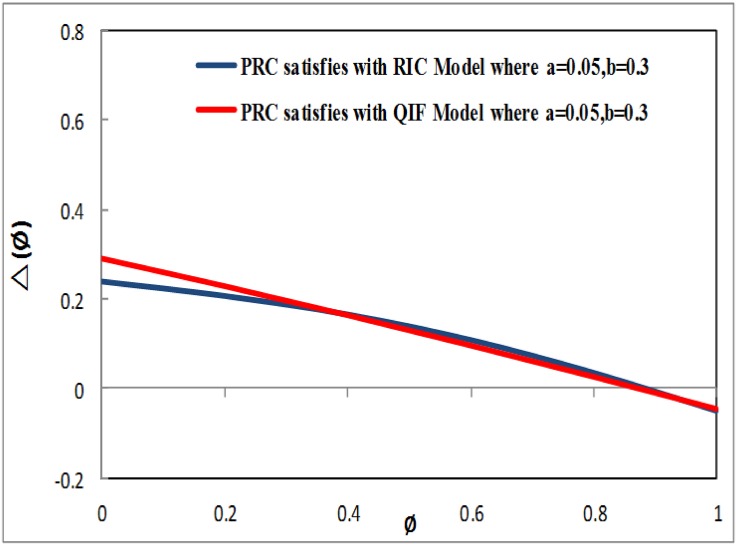
Evaluation of PRC (Δ_*s*_(*ϕ*)) with RIC model and QIF model.

The proof of Eq (1), Δ(*ϕ*_*j*_) in particular, is based on the quadratic integrate and fire (QIF) model [24] and the radial isochron clock model (RIC) [24], and the PRC function satisfies [4], which are developed based on the following models:

- Quadratic Integrate and Fire (QIF) model
  $$\begin{array}{c} \Delta_{QIF}\left(\phi \right)=PRC_{a}\left(1-\cos\left(2\pi \phi \right)\right) \end{array}$$ (2)
  where *PRC*_*a*_ = 0.5, 1.0, −0.5. In this model, an oscillator disregards all stimuli at the time of firing. In addition, it identifies multiple stimuli received simultaneously as one stimulus. For a given value of *PRC*_*a*_, the PRC can both delay and advance the firing. As *PRC*_*a*_ approaches a value of one, the PRC becomes singular. It should be noted that this occurs regardless of the value of a at which the PRC vanishes: *t* = 0, *T*. For the smallest values of *PRC*_*a*_, the QIF of PRC has a particularly simple form.
- Radial Isochron Clock (RIC) model
  $$\begin{array}{c} \Delta_{RIC}\left(\phi \right)=-PRC_{a}*\sin\left(2\pi \phi \right) \end{array}$$ (3)
  where *PRC*_*a*_ = 0.5, 1.0, −0.5. In this model, an oscillator disregards all stimuli at the time of firing, and it also identifies multiple stimuli received simultaneously as one stimulus.
- The PRC function satisfies
  To create a desired TWPCO, regardless of the initial phase, an oscillator must advance its phase towards 1 − *τ* when it is catalysed throughout (0 ≤ *ϕ* < 1 − *τ*). In contrast, its phase must move away from 1 − *τ* when it is catalysed throughout (1 − *τ* < *ϕ* < 1). Therefore, we have the following conditions on the PRC to create a TWPCO:
  $$\begin{array}{c} \left\{ \begin{array}{cc} 0<\Delta (\phi )\leq 1-\tau -\phi & \left(0\leq \phi <1-\tau \right)\\ \Delta (\phi )=0 & \left(\phi =1-\tau \right)\\ 1-\tau -\phi \leq \Delta (\phi )<0 & \left(1-\tau <\phi <1\right) \end{array}\right. \end{array}$$ (4)
  From Eqs (3) and (4), we can generate the following new equation:
  $$\begin{array}{c} \Delta_{s}\left(\phi \right)=-PRC_{a}*\sin\frac{\pi }{1-\tau }*\phi +PRC_{b}\left(1-\tau -\phi \right) \end{array}$$ (5)
  Moreover, from Eqs (2) and (4), Eq (6) is formed as
  $$\begin{array}{c} \Delta_{s}\left(\phi \right)=PRC_{a}*cos\frac{\pi }{2(1-\tau )}*\phi +PRC_{b}\left(1-\tau -\phi \right) \end{array}$$ (6)
  where $PRC_{a}\left(-\frac{PRC_{b}\left(1-\tau \right)}{\pi }<PRC_{a}\leq \frac{\left(1-PRC_{b}\right)*\left(1-\tau \right)}{\pi }\right)$ and 0 < *PRC*_*b*_ ≤ 1 are factors that determine the characteristics of PRC.
  Fig 1 presents the evaluation of PRC (Δ_*s*_(*ϕ*)), which satisfies the RIC model and the QIF model, where *PRC*_*a*_ = 0.1 and *PRC*_*b*_ = 0.5 must be located on the two lines when *τ* = 0.2.
  Fig 2 presents the assessment of PRC (Δ_*s*_(*ϕ*)), which also satisfies the RIC model and the QIF model, where *PRC*_*a*_ = 0.05 and *PRC*_*b*_ = 0.3 must be located on the two lines when *τ* = 0.2.
  By comparing the above evaluations, as shown in Figs 1 and 2, the PRC that satisfies the QIF model in Eq (6) obtained superior results compared to the PRC model that satisfied the RIC model in Eq (5). Our newly generated Eq (6) indicated that the PRC satisfying the QIF model improved the performance of the firefly and pacemaker. The pacemaker may be related to the TWPCO scheme broadcasting the oscillator number of sensor nodes *N*, forwarding the pacemaker information together with a constant offset phase-variation 1 − *τ*.
  As a result, the TWPCO equation obtained from Eq (6) and applied in Eq (1) catalyses the sensor node and modifies its phase as follows:
  $$\begin{array}{c} \phi_{j}=\phi_{j}+PRC_{a}*\cos\frac{\pi }{2*T}*\phi_{j}+PRC_{b}*\left(T-\phi_{j}\right) \end{array}$$ (7)

### 2.2 TWPCO Biological Model

**Fig 3:**
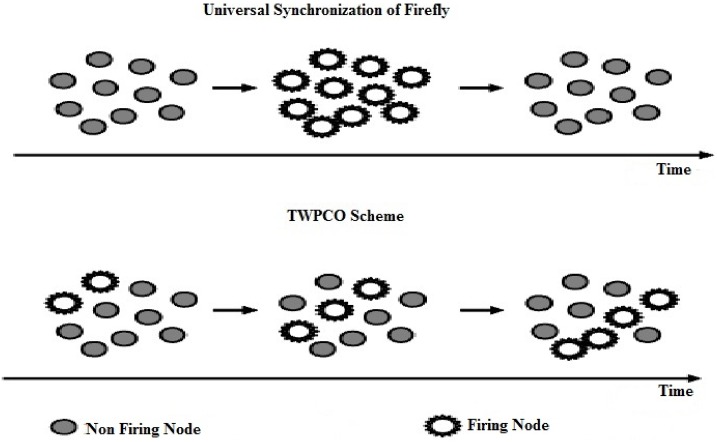
Universal synchronization and TWPCO.

The travelling wave phenomenon can be systematized in data gathering and diffusion based on the biologically inspired network systems of phase locking in the PCO model [2, 7, 32]. The PCO scheme not only exhibits the universal synchronization when all oscillators fire synchronously but also displays the TWPCO, where oscillators behave synchronously with a different fixed phase (Fig 3).

**Fig 4:**
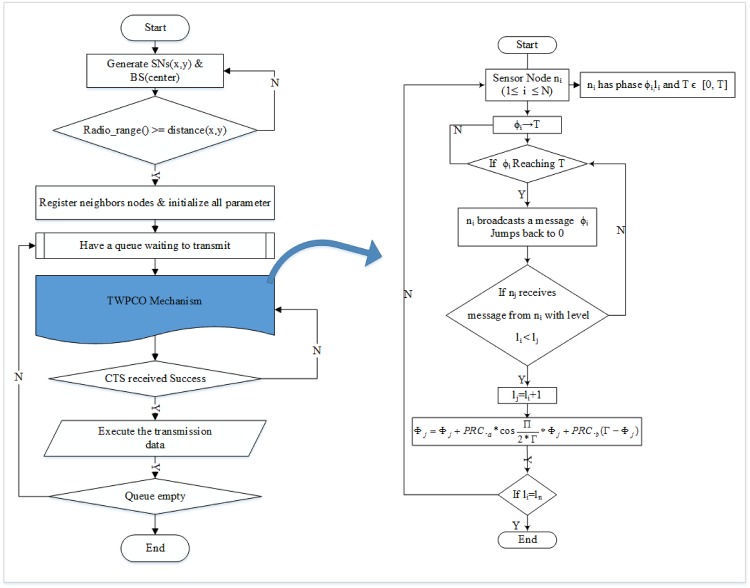
Flowchart of the proposed TWPCO scheme.

In the TWPCO model, each individual sensor broadcasts the gathered data within the phase of its own timer. In this manner, the network adjusts the phase of its own timer accordingly whenever a sensor detects transmissions from another node. By collaborating with their neighbours, sensor nodes eventually enter the phase-locking state in which the sensor data are emitted. In this scenario, the timing of a message being emitted is regarded as a travelling wave phenomenon, whose center occurs at the sensor node and seeks to either gather or disperse information from or to all sensor nodes. Fig 4 outlines the main operations of our developed TWPCO scheme and the flowchart algorithm of the TWPCO scheme.

**Fig 5:**
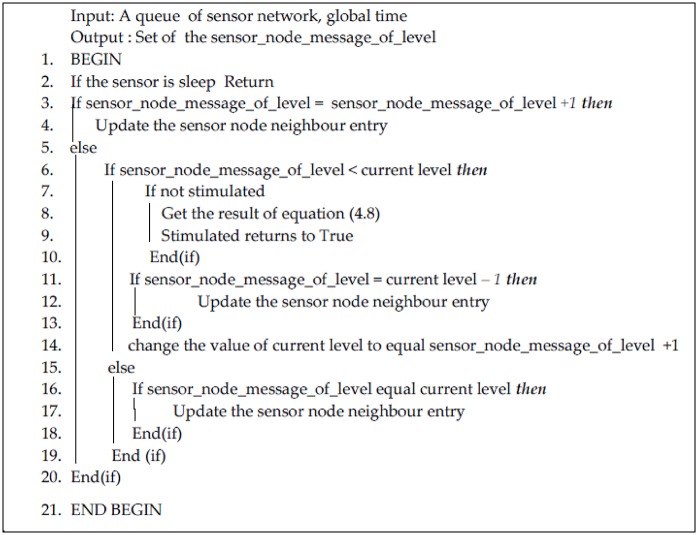
Pseudocode of level adjustment in the TWPCO scheme.

**Fig 6:**
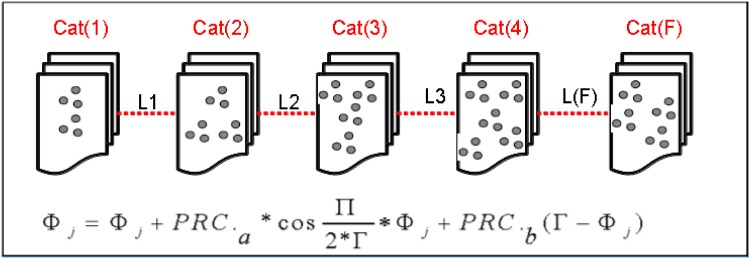
Framework of the TWPCO scheme.

Here, each sensor node *n*_*i*_ (where 1 ≤ *i* ≤ *N*) encompasses the phase timer *ϕ*_*i*_, level *l*_*i*_ as well as the offset *τ*. In the algorithm, the level field is an indication of each sensor’s hop count from the base station. Each sensor’s level field is assigned a large value upon initialization. The offset *τ* is the data transmission interval between the node of level *l* and that of *l*-1. Thus, when the phase *ϕ*_*i*_ reaches *T*, the sensor node *n*_*i*_ sends a broadcast message that contains the control information and sensor data, and then, the phase returns to 0. Upon reception of a message by node *n*_*j*_ from *n*_*i*_ at the level *l*_*i*_ < *l*_*j*_, the node *l*_*j*_ adjusts its level as *l*_*j*_ = *l*_*i*_ + 1. The proposed method is illustrated in full in the pseudocode, which adjusts its level in the TWPCO scheme in Figs 5 and 6, thus stimulating *n*_*j*_ and modifying its phase. According to our TWPCO scheme, the node catalyses and modifies its phase, where *PRC*_*a*_ and *PRC*_*b*_ are the factors that decide the rate of assemblage, as shown in Eq (7). Using Eq (7) in the PRC function, regardless of the initial phases of the nodes, the travelling wave phenomena based on phase-locking in the PCO model are implemented in common communication among sensor nodes.

## 3 Assessment of the Performance of the TWPCO Scheme

In this study, we performed an observation and assessment of the experimental simulation results so that we could evaluate the performance under a variety of WSN environments. According to the experimental results, our scheme plays an important role in enhancing the energy efficiency and data gathering and extending the sensors’ steady states. Such improvement achieved by the TWPCO scheme is attributed to the process of adjusting the quality evaluation of the functions, which as a result, contributes to enhancing the accuracy of the firefly based on the PCO algorithms. To validate the TWPCO scheme, the results of the experiments were compared to the PCO [4]. The next section will explain the experimental setup and assess the accuracy and results of the TWPCO scheme.

### 3.1 Simulation Environment

The suggested TWPCO mechanism was simulated by deploying different sensor nodes. This simulation experiments were performed with a laptop using a simulator implemented in Java. The simulation environment involved a fixed 100*100 m2 square field, with randomly deployed sensor nodes. The BS was located at the center of the field. The transmission radius of the nodes was 50 m, which was assumed to be perfectly omni-directional. Specifically, the MICAz protocol was used in this study. Thus, whenever a node sent two transmission messages from two different nodes simultaneously, the data gathering ratio cycle, *T*, was set to 1 s, while the highest substitute, *t*^*max*^, was set to 0.1*s*. From Eq (7), *PRC*_*a*_ and *PRC*_*b*_ were set to 0.01 and 0.5, respectively. The initial phase readings were randomly set as well. The size of the message header and that of the individual sensor data were set to 2 bytes, whereas that of the message transmission control information was set to 1 byte. Then, the results of the TWPCO scheme were compared with those of the PCO [4]. Moreover, to maintain objectivity, the PCO was re-implemented. This involved using the Java programming language so that both methods could operate or run under the same simulator with similar software and hardware platforms. This was followed by testing and validating the TWPCO method using the same scenarios and simulation setting of the PCO. This was to provide evidence of the efficiency of the TWPCO scheme. A summary of the simulation parameters is presented in Table 1.

**Table 1:** Parameter Setup.

Parameters	Values	
	Scenario I	Scenario II
Channel Frequency	2.4 GHz	2.4 GHz
Number of Nodes	10.20, 30, 40, 50, 60, 70, 80, 90, 100	30
MIN_TIME_STEP	0.00001	0.00001
Packet Data Size	16 bits	8.16, 40, 80, 160, 400, 800 bites
Energy Model	MICAz	MICAz
Data Rate	250 kbps	250 kbps
Transmit Power	52.2 *μ*W	52.2 *μ*W
Receive Power	59.1 *μ*W	59.1 *μ*W
Idle Power	60 *μ*W	60 *μ*W
Sleep power	3 *μ*W	3 *μ*W
INITIAL_ENERGY	100 Joules	100 Joules

### 3.2 Evaluation of the Performance Metrics

This section is concerned with the calculation and benchmarking of the performance metrics of the proposed scheme with metrics of similar schemes. Similar to the other schemes for achieving energy efficiency, the performance of our TWPCO scheme was measured in relation to two significant effects: the effect of the number of sensor nodes and the effect of the packet size. This scheme mainly focuses on improving the system performance by reducing the energy and extending the lifespan of the network. The performance metrics that are discussed in this section of the paper are the transient steady state, data gathering ratio, and energy efficiency ratio.

#### 3.2.1 Evaluation of Transient and Steady States

The study evaluated the fundamental performance of the proposed mechanisms in the transient state. The results showed that the WSN started with the sink and five sensor nodes, from 1 to 5, at cycle = 0 in the simulation. Then, the addition of sensor nodes 6 and 7 at cycle = 40 was performed, and at cycle = 70, sensor nodes 1 and 4 were removed from the WSN to confirm the self-organization feature of the WSN. The initial phase values of the sensor nodes were randomly set.

#### 3.2.2 Data Gathering Ratio

The data gathering ratio is the ratio of the overall sensor node data collected at the sink per cycle. This ratio is calculated as follows:

$$\begin{array}{c} DataGathering=\frac{\sum_{i=1}^{n}Topck_{sink}}{SNC} \end{array}$$ (8)

where n represents the number of sensor nodes in the network and *Topck*_*sink*_ is the total number of packets that are received by the sink node for each sensor node i. Moreover, SNC is the number of sensor nodes in each cycle.

#### 3.2.3 Energy Efficiency Ratio

The energy efficiency ratio is the ratio of the overall consumption of energy to the number of packets that are received by the core node. This ratio is calculated as follows:

$$\begin{array}{c} EnergyEfficiency=\frac{\sum_{i=1}^{n}ToEnc\left(i\right)}{Topck_{sink}} \end{array}$$ (9)

where n represents the number of sensor nodes in the network, ToEnc(i) is the total energy consumption for each sensor node i, and *Topck*_*sink*_ is the total number of packets received by the sink node.

### 3.3 Discussion of Experimental Results

This section discusses the main experimental results of the current study, with which we validated the efficiency of our proposed TWPCO scheme in comparison to the PCO. These results were obtained using the Java programming language. The results discussed here focus on three main aspects: evaluation of the transient and steady state, the impact of the number of sensor nodes and the impact of packet size on the assessed accuracy of the proposed scheme.

#### 3.3.1 Evaluation of Transient and Steady State

This sub-section discusses the results of the main experiments (see sub-section: Evaluation of Transient and Steady States) regarding the evaluation of the transient and steady state.

**Fig 7:**
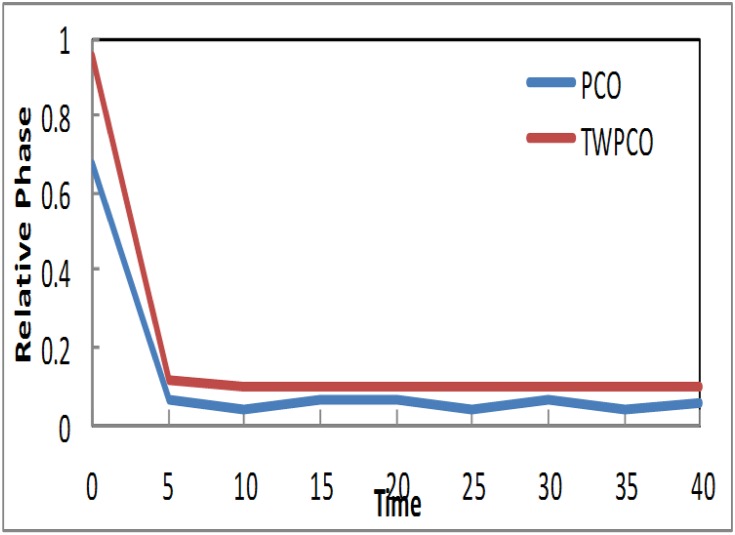
Relative Phase of Sensor TWPCO and PCO in regard to Node 1.

**Fig 8:**
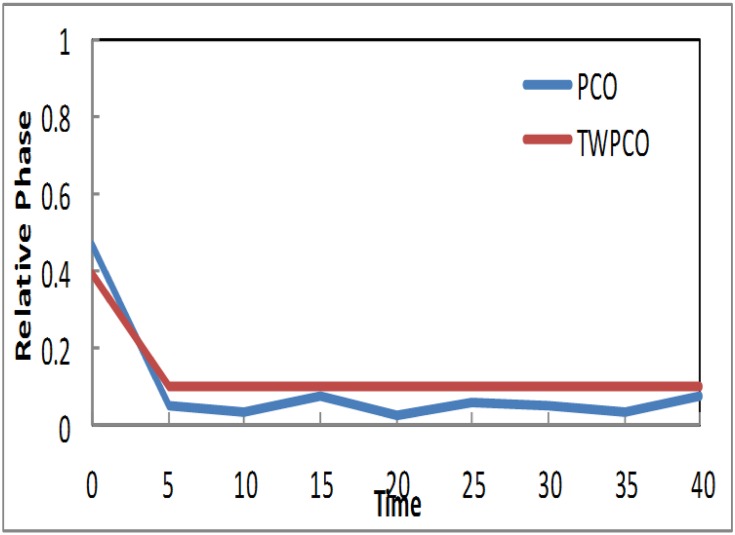
Relative Phase of Sensor TWPCO and PCO in regard to Node 2.

**Fig 9:**
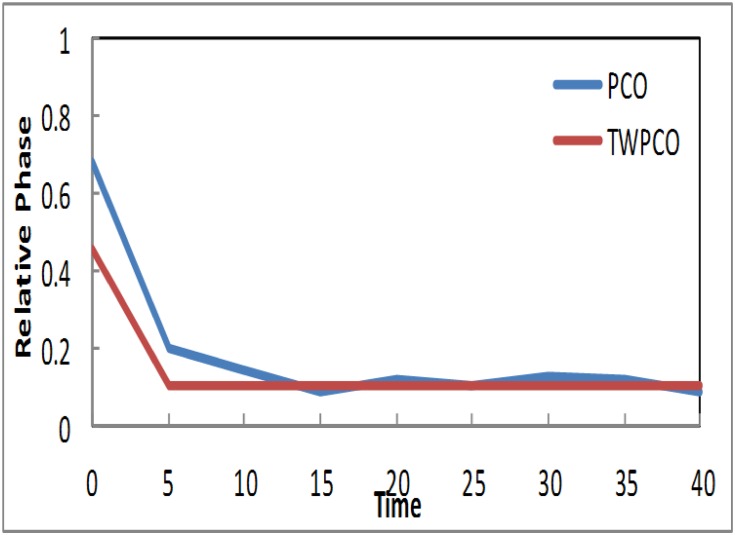
Relative Phase of Sensor TWPCO and PCO in regard to Node 4.

Based on the results in Figs 7–9, in our TWPCO scheme, the sensor nodes (1, 2 & 4) took almost 5 cycles to reach the steady state. This steady state is due to the application of the TWPCO mathematic model and specifically because of the new equation used in this study. In contrast, in the PCO, the sensor nodes (1, 2 & 4) did not reach any steady state in all cycles. Such results indicate that our proposed scheme outperformed the PCO in relation to the steady state reached by the nodes. This is because the mechanism of the proposed model was stronger than that of the PCO model.

**Fig 10:**
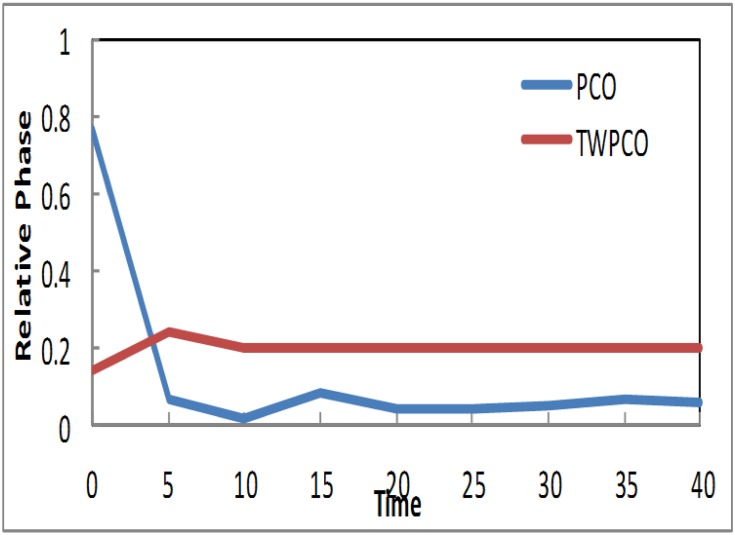
Relative Phase of Sensor TWPCO and PCO in regard to Node 3.

**Fig 11:**
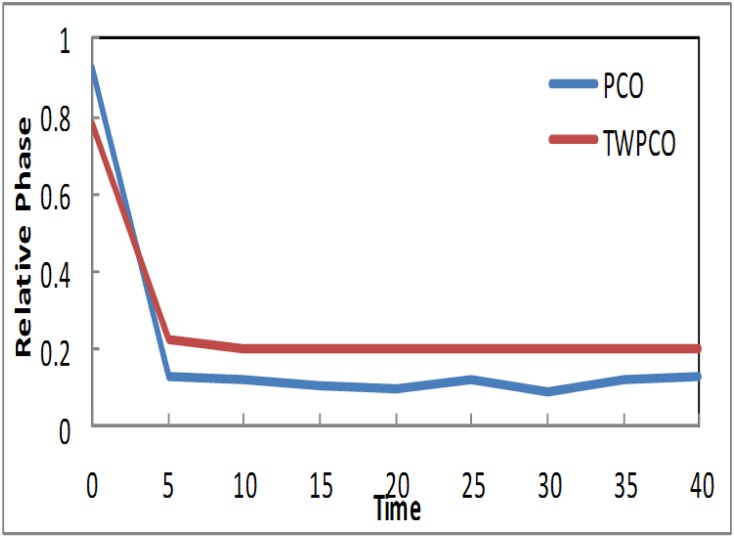
Relative Phase of Sensor TWPCO and PCO in regard to Node 5.

Similarly, the results in Figs 10 and 11 show that, in the TWPCO scheme, node sensors 3 and 5 took almost 10 cycles to attain steady state. Although node sensors 1, 2 and 4 took 5 cycles to achieve this steady status, node sensors 3 and 5 attained this status at 10 cycles. This difference is due to the different levels of the two categories of nodes, as previously mentioned in Section. However, in the PCO, nodes 3 and 5 did not attain such a steady state in all cycles. Therefore, it can be concluded that, in general, the TWPCO scheme achieves a better cycle performance than does the PCO scheme in terms of the nodes reaching steady state. This better performance of the proposed model is attributed to the advantage of the TWPCO scheme in enabling the nodes to more easily attain this steady state. This is one of the major contributions of the present study.

**Fig 12:**
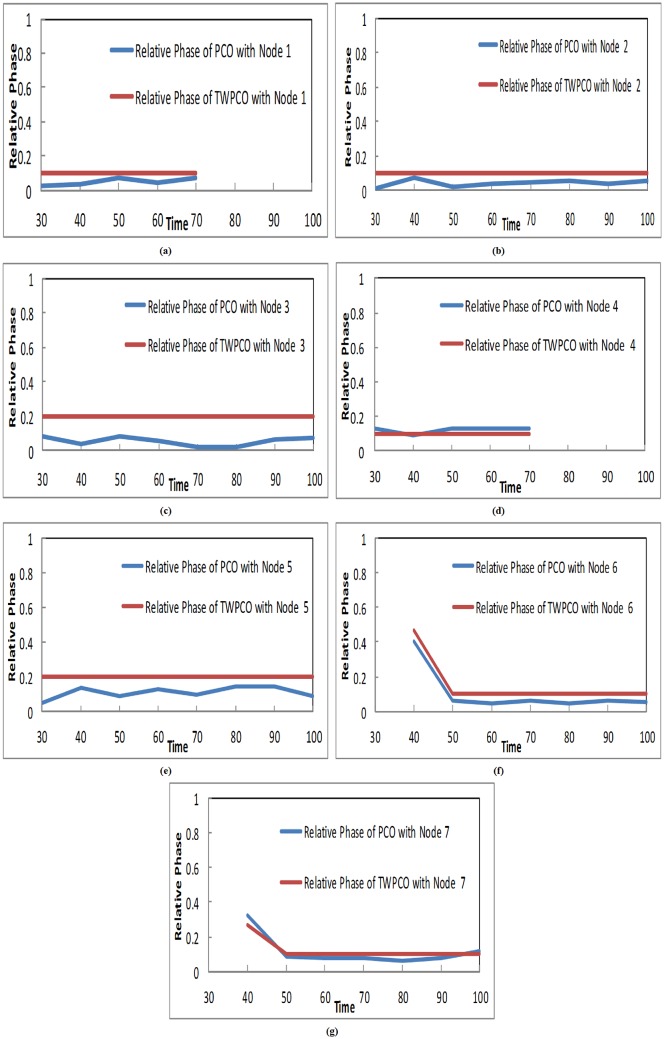
Evaluation of Relative Phase under the TWPCO and PCO Schemes for Sensor Nodes against the Addition and Removal of Sensor Nodes.

Fig 12 illustrates the relative phase of the sensor nodes against the removal and addition of sensor nodes. It is apparent that, in the proposed TWPCO scheme, nodes 6 and 7 were added to the WSN at the 40th cycle with random initial phases. The nodes’ transmission timing eventually reached a steady state. Similarly, nodes 1 and 4 were removed from the network at the 70th cycle. These mentioned nodes were added and removed to confirm the self-organization feature of the WSN.

Fig 12a-12g illustrates an evaluation of the relative phases of the sensor nodes against the addition/removal of nodes under the proposed model compared to the PCO. This illustrates that the transient of the steady state of the TWPCO scheme reaches steady state after a number of cycles compared to the PCO. Thus, our scheme obtained superior results compared to the PCO. The superior results reported in this study are a result of the suitability and self-organization of our TWPCO scheme.

#### 3.3.2 The Impact of the Number of Sensor Nodes on the Assessed Accuracy of the TWPCO Scheme

**Fig 13:**
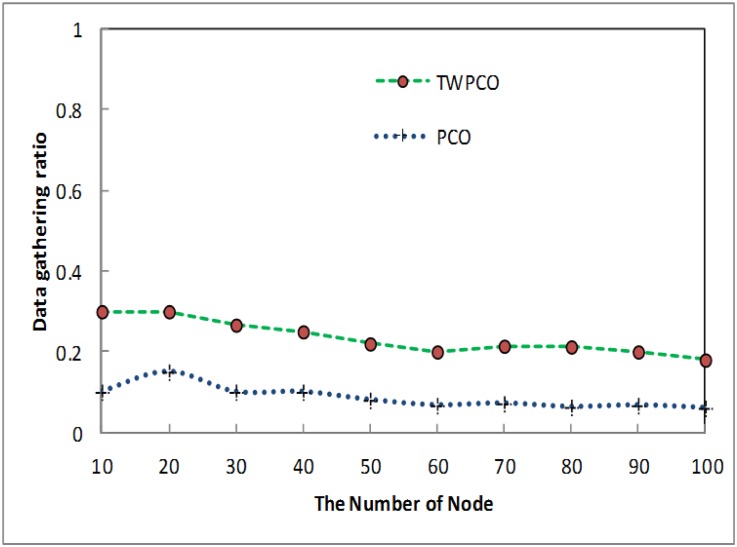
Data gathering ratio based on number of nodes.

From scenario I (Table 1 and Fig 13), the data gathering ratio of the TWPCO scheme was compared to that of the PCO. Fig 13 demonstrates that, in both mechanisms, there is a degradation of the data gathering performance with increasing number of sensor nodes. In scenario I, the data gathering ratio of the TWPCO scheme declined as the number of the sensor nodes exceeded 40. This declining data gathering ratio could be due to the simulation results. Although the data gathering performance seemed to decrease, the TWPCO scheme still outperformed the PCO because the techniques used in our proposed scheme are more useful than those of the PCO. Specifically, evaluation of the TWPCO scheme in comparison with the PCO revealed that the TWPCO method is up to 9% superior to the PCO in terms of data gathering. In addition, the packet size of the TWPCO scheme is larger than that of the PCO. This is because the packet broadcast timing information was included in the TWPCO model. This improvement of the TWPCO mechanism is attributed to the fact that the TWPCO scheme uses the new mathematical model for calculating the hidden node problem.

**Fig 14:**
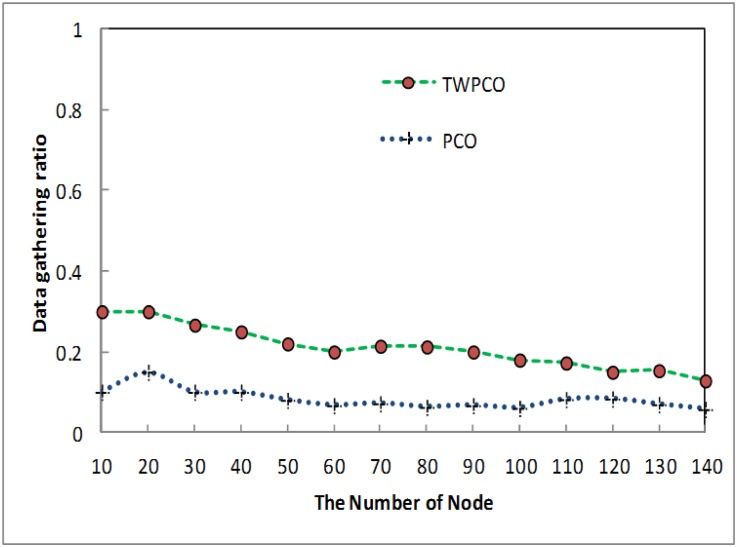
Data gathering ratio based on the number of nodes added and the number of sensor nodes.

Fig 14 shows the gathering data ratio of the TWPCO scheme as compared to the PCO. From this Fig, it is apparent that, in the proposed TWPCO scheme, sensor nodes 110, 120, 130 and 140 were added to the WSN in scenario I (Table 1). With these sensor nodes, the TWPCO scheme still outperformed the PCO in terms of the data collection ratio. This indicates that the increasing number of nodes did not affect the performance of our proposed scheme. The performance of the TWPCO scheme for sensor nodes 110-140 was estimated to be 5% superior to the PCO in relation to data gathering.

**Fig 15:**
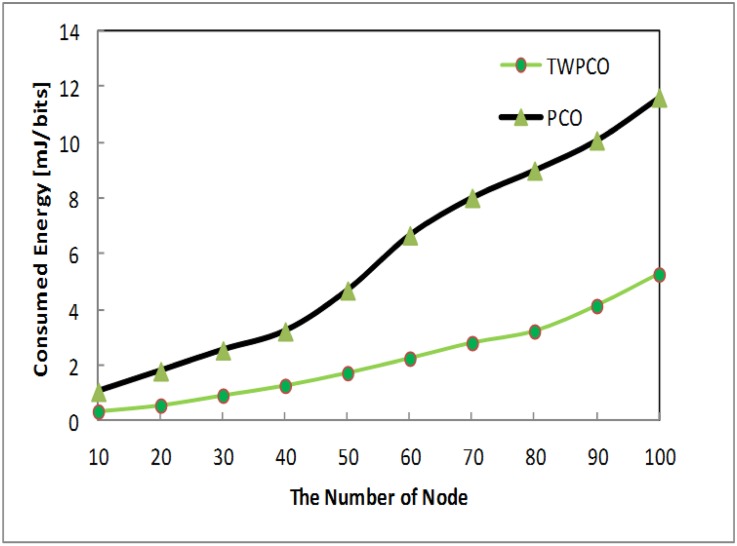
Energy efficiency ratio based on the number of nodes.

Fig 15 shows that the performance of the TWPCO scheme was superior to the PCO in terms of energy efficiency. This is due to the higher number of received packets in the TWPCO scheme, which made the scheme more efficient. Specifically, the TWPCO scheme exhibits a 25% higher energy savings performance compared to the PCO. As a result, the energy efficiency ratio of the TWPCO scheme is reduced significantly compared to the PCO, thus demonstrating superior results.

**Fig 16:**
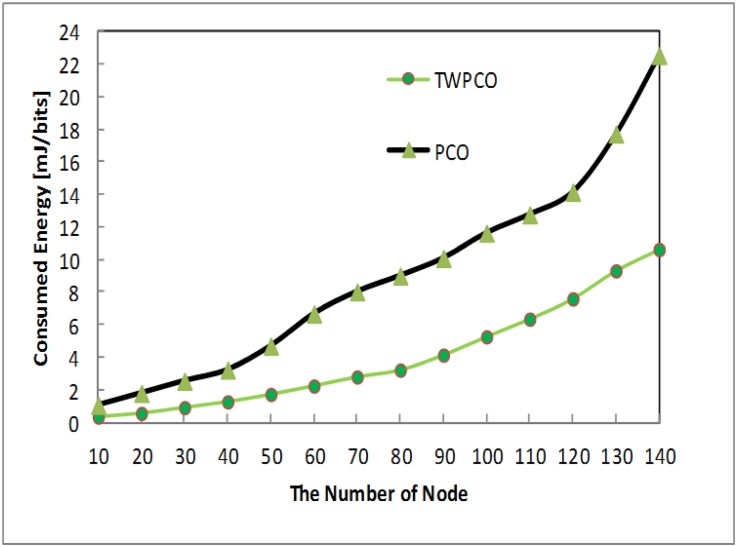
Energy efficiency ratio based on the number of nodes and the added number of sensor nodes.

Fig 16 shows the energy efficiency ratio of the TWPCO scheme compared to the PCO. From this Fig, it is apparent that, in the proposed TWPCO scheme, sensor nodes 110, 120, 130 and 140 were added to to the WSN under scenario I (Table 1). With these sensor nodes, the TWPCO scheme also obtained superior results in comparison to the PCO in terms of the energy efficiency ratio. This implies that our proposed scheme tolerates the increasing number of received nodes. Specifically, the TWPCO scheme at nodes 110-140 achieves a 20% higher energy savings performance than does the PCO.

#### 3.3.3 The Impact of the Packet Size on the Assessed Accuracy of the TWPCO Scheme

**Fig 17:**
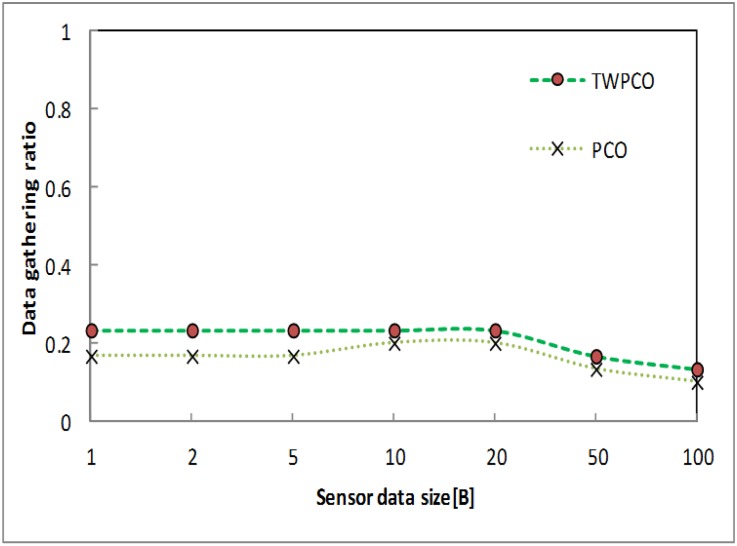
Data gathering ratio based on data packet size.

**Fig 18:**
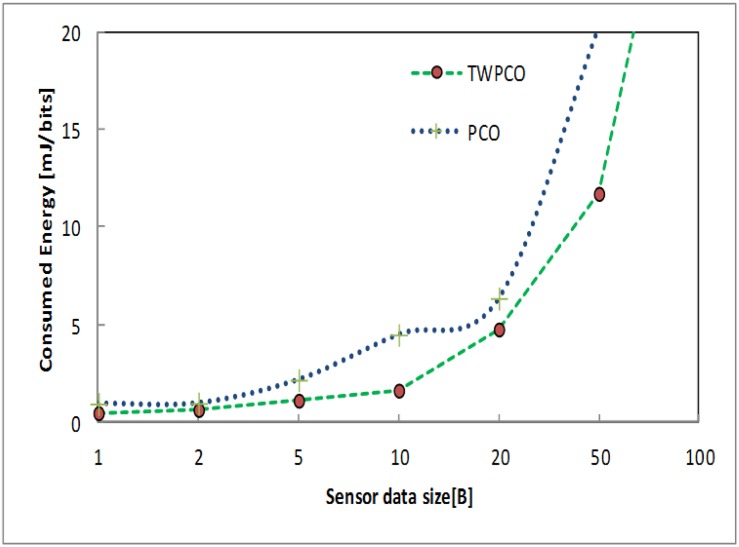
Energy efficiency ratio based on data packet size.

Under scenario II (see Table 1), Figs 17 and 18 illustrate the data gathering and energy efficiency per sensor node datum size for both schemes. The data gathering ratio of the TWPCO scheme was higher than that of the PCO (Fig 17). This is one of the contributions of our proposed scheme in this study. This improvement is because of the categories of the levels of the nodes. The TWPCO scheme achieved a 9% improvement in the data gathering ratio compared to the PCO. On the other hand, the energy efficiency ratio of the TWPCO scheme declined considerably compared to the PCO (Fig 18). The decreasing ratio of the energy efficiency of the PCO is an indicator of the weaknesses of this scheme.

**Fig 19:**
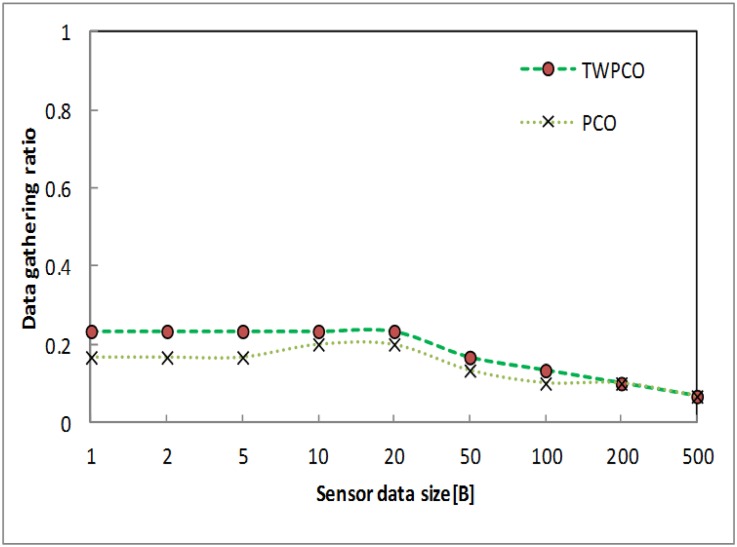
Data gathering ratio based on data size and added data size.

**Fig 20:**
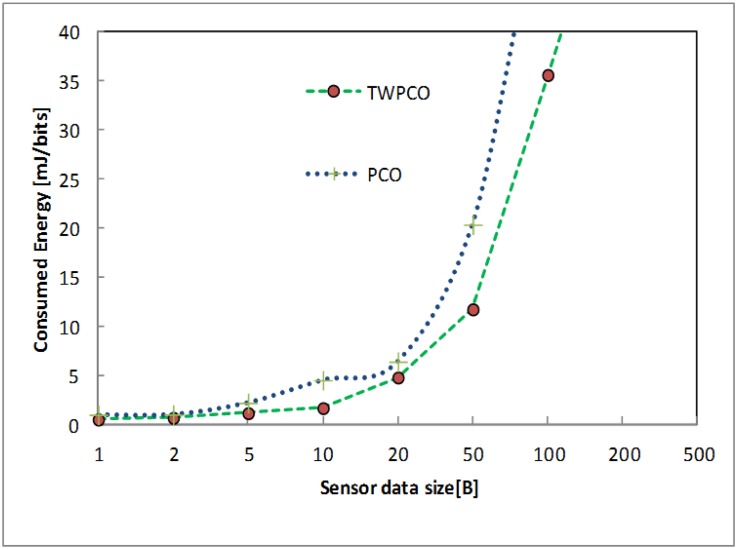
Energy efficiency ratio based on data size and added data size.

Figs 19 and 20 show the energy efficiency ratio and data collection ratio of the TWPCO scheme compared to the PCO. According to these two Figs, in the proposed TWPCO scheme, the packet sizes of 200 and 500 were added to the WSN under scenario II (Table 1). With these packet sizes, the TWPCO scheme’s data gathering ratio was equal to that of the PCO when exceeding 200 bytes, as shown in Fig 19. This equal ratio of data gathering achieved by the two schemes is attributed to the limited memory of the sensor nodes. On the other hand, the TWPCO scheme’s energy efficiency ratio was too high compared to the PCO when exceeding 200 bytes, as shown in Fig 20. This is due to the size of the data packet of the sensor nodes. Thus, our scheme still outperformed the PCO in terms of energy efficiency ratio and data collection ratio.

Summarizing, regardless of the sensor node data size, the TWPCO scheme achieves an up to 13% increase in performance in comparison to the PCO in terms of both the energy consumption ratio and the data gathering ratio (Figs 13 to 20). Therefore, the TWPCO model, for mimicking the flashing synchronization behaviour of fireflies and the emission of radio signals as firing, is suited for WSN applications with a high data gathering ratio and energy efficiency ratio for mitigating the issue of deafness. Based on the above results, the application of mathematical and biological models of the TWPCO scheme to WSNs provided evidence of the suitability and strength of this scheme.

## 4 Conclusions

This paper proposed a travelling wave phenomena algorithm based on the phase-locking of the PCO for WSNs. The model mimics the synchronization behaviour of fireflies and the emission of radio signals as firing. To compare the performance of the proposed model with an existing standard, the model was compared with PCO in terms of the data gathering as well as the energy efficiency ratio. Using simulations, the results confirmed that the TWPCO scheme outperformed the PCO in all aspects of the comparison and also confirms the suitability of the proposed mechanism for WSNs. However, future research should confirm and improve the performance of these mechanisms using the reachback of firefly algorithm (RFA) in an experimental evaluation as well as an actual sensing environment.

## Supporting Information

S1 Code This is the S1 Code title. (ZIP)
