# Supplementary material for: Travelling Wave Pulse Coupled Oscillator (TWPCO) Using a Self-Organizing Scheme for Energy-Efficient Wireless Sensor Networks
Source: PLoS One. 2017 Jan 5;12(1):e0167423. doi: 10.1371/journal.pone.0167423 (PMC5215802; doi:10.1371/journal.pone.0167423)
Supplement: S1 Code — (ZIP) [file pone.0167423.s001.zip › code/src-basic/doc/index.html]

Generated Documentation (Untitled)


<H2>
Frame Alert</H2>
<P>
This document is designed to be viewed using the frames feature. If you see this message, you are using a non-frame-capable web client.
<BR>
Link to<A HREF="Simulation.html">Non-frame version.</A>
