# Supplementary material for: Travelling Wave Pulse Coupled Oscillator (TWPCO) Using a Self-Organizing Scheme for Energy-Efficient Wireless Sensor Networks
Source: PLoS One. 2017 Jan 5;12(1):e0167423. doi: 10.1371/journal.pone.0167423 (PMC5215802; doi:10.1371/journal.pone.0167423)
Supplement: S1 Code — (ZIP) [file pone.0167423.s001.zip › code/src-basic/doc/package-frame.html]

<Unnamed>


<Unnamed>

|  |
| --- |
| Classes    Simulation |
