# Supplementary material for: Travelling Wave Pulse Coupled Oscillator (TWPCO) Using a Self-Organizing Scheme for Energy-Efficient Wireless Sensor Networks
Source: PLoS One. 2017 Jan 5;12(1):e0167423. doi: 10.1371/journal.pone.0167423 (PMC5215802; doi:10.1371/journal.pone.0167423)
Supplement: S1 Code — (ZIP) [file pone.0167423.s001.zip › code/src-basic/doc/package-summary.html]

---


---

## Package <Unnamed>

| **Class Summary** | |
| --- | --- |
| **Simulation** |  |

---


---
