# Supplementary material for: Travelling Wave Pulse Coupled Oscillator (TWPCO) Using a Self-Organizing Scheme for Energy-Efficient Wireless Sensor Networks
Source: PLoS One. 2017 Jan 5;12(1):e0167423. doi: 10.1371/journal.pone.0167423 (PMC5215802; doi:10.1371/journal.pone.0167423)
Supplement: S1 Code — (ZIP) [file pone.0167423.s001.zip › code/src-basic/doc/Simulation.html]

Simulation


---


---


## Class Simulation

```
java.lang.Object
  Simulation
```

---

``` public class Simulation extends java.lang.Object ```

---

| **Field Summary** | |
| --- | --- |
| `(package private)  boolean` | `enable_csma` |
| `(package private)  double` | `global_time` |
| `(package private)  Network` | `network` |
| `(package private)  int` | `node_num` |
| `(package private)  Parameters` | `param` |
| `(package private)  double` | `radio_range` |
| `(package private)  java.util.Random` | `rand` |
| `(package private)  long` | `rseed` |
| `(package private)  double` | `simulation_duration` |
| `(package private)  double` | `tau_max` |
| `(package private)  int` | `used_method` |


| **Constructor Summary** | |
| --- | --- |
| `Simulation(java.lang.String[] args)` |


| **Method Summary** | |
| --- | --- |
| `void` | `do_simulation()` |
| `static void` | `main(java.lang.String[] args)` |

| **Methods inherited from class java.lang.Object** |
| --- |
| `clone, equals, finalize, getClass, hashCode, notify, notifyAll, toString, wait, wait, wait` |

| **Field Detail** |
| --- |

### enable\_csma

```
boolean enable_csma
```

---


### global\_time

```
double global_time
```

---


### network

```
Network network
```

---


### node\_num

```
int node_num
```

---


### param

```
Parameters param
```

---


### radio\_range

```
double radio_range
```

---


### rand

```
java.util.Random rand
```

---


### rseed

```
long rseed
```

---


### simulation\_duration

```
double simulation_duration
```

---


### tau\_max

```
double tau_max
```

---


### used\_method

```
int used_method
```


| **Constructor Detail** |
| --- |

### Simulation

```
public Simulation(java.lang.String[] args)
```


| **Method Detail** |
| --- |

### do\_simulation

```
public void do_simulation()
```

---


### main

```
public static void main(java.lang.String[] args)
```


---


---
